# Radial-tangential mode of single-wall carbon nanotubes manifested by Landau regulation: reinterpretation of low- and intermediate-frequency Raman signals

**DOI:** 10.1038/s41598-023-32018-4

**Published:** 2023-03-27

**Authors:** K. P. S. S. Hembram, Jin-Gyu Kim, Sang-Gil Lee, Jeongwon Park, Jae-Kap Lee

**Affiliations:** 1 Center for Opto-Electronic Materials and Devices, Korea Institute of Science and Technology (KIST), Seoul, 02792 Republic of Korea; 2 Center for Research Equipment, Korea Basic Science Institute, Daejeon, 34133 Republic of Korea; 3 Department of Electrical and Biomedical Engineering, University of Nevada, Reno, NV 89557 USA; 4 School of Electrical Engineering and Computer Science, University of Ottawa, Ottawa, ON K1N 6N5 Canada

**Keywords:** Materials science, Nanoscience and technology, Physics

## Abstract

The low-frequency Raman signals of single-wall carbon nanotubes (SWNTs), appearing in the range of 100–300 cm^−1^, have been interpreted as radial-breathing mode (RBM) comprising pure radial Eigenvectors. Here, we report that most of the low-frequency and intermediate-frequency signals of SWNTs are radial-tangential modes (RTMs) coexisting radial and tangential Eigenvectors, while only the first peak at the low-frequency side is the RBM. Density functional theory simulation for SWNTs of ~ 2 nm in diameter shows that dozens of RTMs exhibit following the RBM (~ 150 cm^−1^) up to *G*-mode (~ 1592 cm^−1^) in order with Landau regulation. We specify the RBM and the RTM on Raman spectra obtained from SWNTs, where both appear as prominent peaks between 149 and 170 cm^−1^ and ripple-like peaks between 166 and 1440 cm^−1^, respectively. We report that the RTMs have been regarded as RBM (~ 300 cm^−1^) and ambiguously named as intermediate-frequency mode (300–1300 cm^−1^) without assignment. The RTMs gradually interlink the RBM and the *G*-mode resulting in the symmetric Raman spectra in intensity. We reveal high-resolution transmission microscope evidence for a helical structure of SWNTs, informing the typical diameter of commercial SWNTs to be 1.4–2 nm.

## Introduction

Raman spectra of single-wall carbon nanotubes (SWNT)^1^ exhibit the tangential *G*-mode at ~ 1592 cm^−1^ (including *G*^-^ peak at 1572 cm^−1^) and the non-tangential low-frequency signals at 100–300 cm^−1^, together with the defect-related *D* band at ~ 1350 cm^−1^. The low-frequency signals have been interpreted as radial-breathing mode (RBM)^2^, which is regarded as an important indicator of discerning diameters as well as electronic properties of SWNTs^1–6^. This is due to the general understanding that it originated with synchronous radial vibration of the carbon atoms^2,3^ in tubular graphene to be correlated with their diameters, affecting, in turn, their chirality^7^. The RBM mostly appears as a band comprising multiple peaks, and this band signal has been attributed to the presence of plural SWNTs with different diameters to be ~ 0.5–2 nm for 100–300 cm^−1^ from the equation, ω_RBM_ (cm^−1^) =  ~ 248/*d* where *d* is diameters of SWNTs^3–5^.

The low-frequency Raman signals have also been observed from graphene (or nano-graphitic) structures^8–16^. This is unexpected because they are considered unique to SWNTs, *i.e*., tubular graphene. Thus some researchers name the low-frequency Raman signals of the graphene structures as RBM-like without further explanation^11,15^ or layer breathing modes of bilayer graphene^13,14^. Lee et al. have shown that the low-frequency Raman signals of graphene structures (100–500 cm^−1^) are due to the radial mode (RM) formed by end nano-curvatures of mono- or bilayer graphene^16^. Indeed, the limit of the low-frequency Raman signals of SWNTs has been inconsistently reported to be ~ 200, ~ 250, ~ 300, ~ 350, and ~ 400 cm^−1^, while some well-defined Raman spectra of SWNTs reveal many ripple-like peaks^1–5,17–27^ distributed from ~ 200 cm^−1^ to ~ 1300 cm^−1^. Those appearing between ~ 600 and ~ 1300 cm^−1^ have been generally named as intermediate-frequency modes (IFM)^1,2,18,19,23–27^ without assignment or overlooked^3–5,21,22^. This IFM naming (which means ‘unassigned’) left another issue, the unclear boundary between RBMs and IFMs. Also, the calculated diameters of 0.5–2.0 nm for 100–500 cm^−1^ are too small, compared with 1.4–2 nm of SWNTs directly observed by HRTEM^21,28–33^. These indicate that the nature of the vibrational modes still puzzles in spite of numerous investigations for the different graphene structures.

Recently, there has been an issue with the structure of SWNTs, *i.e*., to be non-tubular (helical) morphology, which is contrary to the conventional tube model (Supplementary Fig. 1). High-resolution transmission microscope (HRTEM)^28–33^ and scanning tunneling microscope (STM)^34,35^ images where traces of graphene helix are evident (Supplementary Fig. 2), energy calculation^28^, as well as direct field emission microscopy observation where the turning (*i.e*., helical) growth of SWNTs^36^ have all been used to support the helix model. However, these evidences for helical SWNTs have yet to be noticed or overlooked with the chiral theory^7^, which not only provides theoretical background of the tube model but also justifies the diverse electronic features^6,23^, conducting or semiconducting with a different band gap of SWNTs reported^35^.

Park et al. have reinterpreted SWNTs as a graphene helix with the Raman study, including simulations for different (concentric and opened) SWNT structures^37^. They have shown that the *G*^*-*^ peak (~ 1575 cm^−1^) unique to SWNTs is the characteristic of their helical structure, reporting the radial-tangential modes (RTMs) assigned as the shoulder peak (~ 186 cm^−1^) of the RBM. In this article, we explore vibrational features for concentric and opened (helical) tube structures as well as curved graphene with first-principles calculation and measurement and show that RBM, RTM, and *G*-mode are governed by Landau regulation.

## Results and discussion

### TEM and Raman analysis

**Figure 1:**
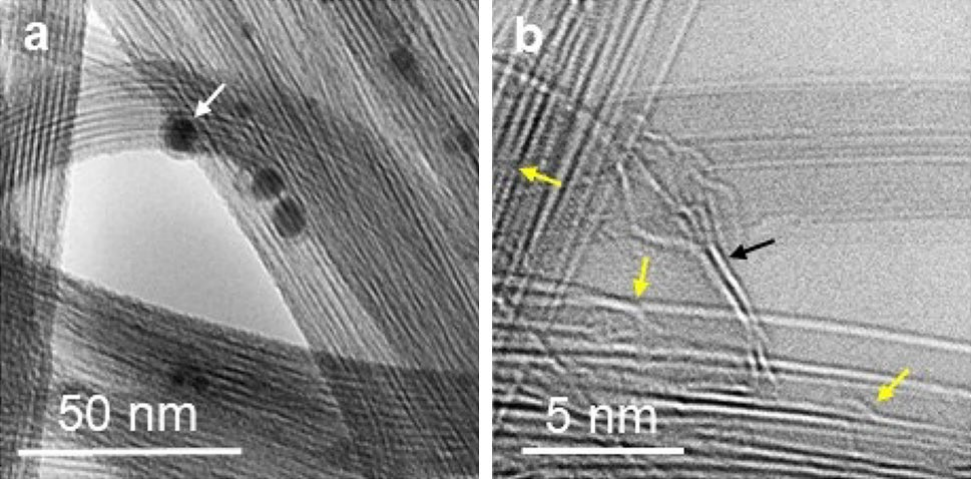
TEM images for Tuball-SWNTs. (**a**) Low magnification. (**b**) HRTEM. The samples coexist with metal catalysts (white arrow in (**a**)) as well as graphitic structures (black arrow in (**b**)).

Figure 1 shows TEM images for Tuball-SWNTs. They appear as a bundle of tens of nm in diameter with a metal catalyst (white arrow in Fig. 1a), which is the same as those for the samples reported by others^37^. Diameters are measured to be ~ 1.4–2.0 nm, similar to those of the arc-SWNT samples^28^ (Fig. 1b), and some samples reveal helical traces indicated by the yellow arrows.

**Figure 2:**
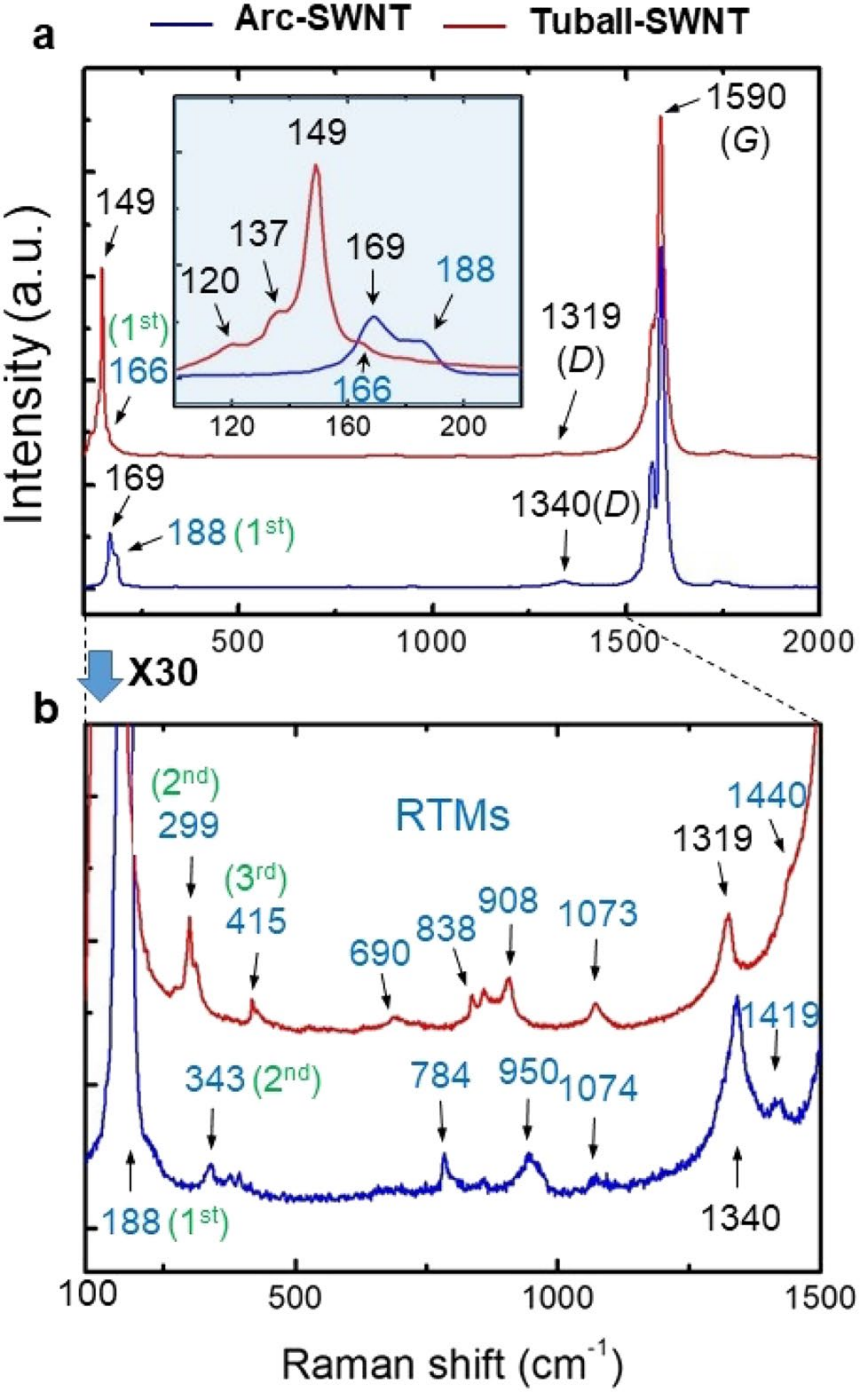
Raman spectra of SWNT samples. (**a**) Raman spectra obtained from purified and bundled Tuball- and arc-SWNTs samples revealing typical Raman signals for SWNTs. Inset in (**a**) shows a zoom-in view of the low-frequency range. (**b**) Zoom-in view of the spectra of (**a**).

**Figure 3:**
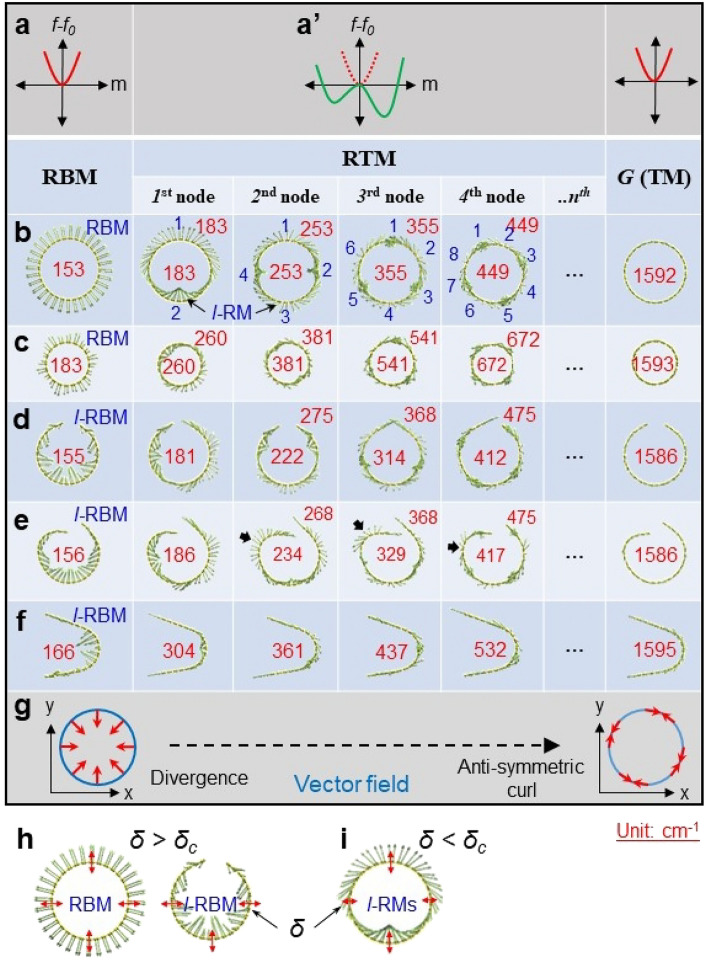
Simulated Raman-active modes for different graphene structures. (**a**, **a**’) Landau free energy landscape with one (**a**) or two minima (**a**’), which are the conditions of RBM (*l*-RBM) and *G*-mode or RTMs, respectively. (**b**–**f**), Raman modes simulated from concentric tubes of ~ 2.2 nm (**b**) and 1.4 nm (**c**) in diameter, opened tubes (**d**,**e**) and curved graphene (**f**), revealing RBM (or *l*-RBM), RTM and *G*-mode in order. We used the opened tubes instead of helical structures. The blue numbers represent the number of nodes for each RTM. The red numbers in (**b**–**f**) represent the frequencies of the modes. (**g**) Diagrams explaining divergence and anti-symmetric curl of vector fields. (**h**, **i**) Schematic explaining Landau condition for RBM (*δ* > *δ*_*c*_) (**h**) and RTM (*δ* < *δ*_*c*_) (**i**).

Raman spectra obtained from the two kinds of samples reveal the low-frequency band of 100–200 cm^−1^ (inset), the *D* band at ~ 1350 cm^−1^, and the strong tangential *G* band at ~ 1590 cm^−1^, as shown in Fig. 2a, which are typical to SWNTs^1–5^. Arc-SWNTs reveal a strong peak at 169 cm^−1^ and its shoulder peak at 188 cm^−1^, while Tuball-SWNTs reveal a strong peak at 149 cm^−1^ and a few peaks at 120 cm^−1^, 137 cm^−1^, and 166 cm^−1^. Both samples reveal many weak but clear signals at ~ 200–1500 cm^−1^, as shown in their magnified Raman spectra (Fig. 2b). We assign these ripple-like peaks to the RTMs where low nodes (1st, 2nd, and 3rd) peaks are specified with the simulated Raman modes for concentric and opened tube structures shown in Fig. 3.

### Raman simulation

The RBM, where all Eigenvectors are directed towards the center of the axis, is evident in the 0th node of the concentric tube (Figs. 3b and c). The 0th node for the opened tube (helical geometry) and curved graphene reveals localized RBM (*l*-RBM) where the radial Eigenvectors are localized (Fig. 3d–f) compared with that of RBM. RTMs, featured by a mixture of localized radial mode (*l*-RM) and tangential mode (*l*-TM) Eigenvectors, reveal two degenerated or non-degenerated frequencies per node in between RBM and *G*-mode (Supplementary Fig. 3). The total number of RTMs (*N*_RTM_) for the concentric tube is 2*n* where *n* is the number of nodes, and ‘2’ represents two modes per node. Two modes in each node for the concentric tube are degenerate, while those for the opened tube are non-degenerate. We attribute the non-degeneracy, the appearance of the *l*-RBM (or *l*-RM), and the lack of one 1^st^ node RTM for the opened tube, to their structural asymmetry (Fig. 3d and e).

The locally distorted opened SWNT structure reveals relatively strong *l*-RMs on the steep edge curvature at higher nodes up to the 4^th^ (Supplementary Fig. 3). Frequencies of RBMs (*l*-RBM) and RTMs for SWNTs depend on their diameters as well as local curvatures. With an increase of nodes, the frequencies of RTMs monotonically increase, and the *l*-RMs become weaker. The simulation data indicates that the *G*-mode and the RBM are both extreme Raman modes comprising pure RM and pure TM Eigenvectors, respectively, with an energy state (Fig. 3).

### Landau regulation of Raman modes

We see the *l*-RM and the *l*-TM comprising the RTMs to two different phases in a phase diagram of a material where Landau theory works^39^. This enables us to clarify the simulation data shown in Fig. 3 with Landau free energy formula, *f*(*δ*) = *f*_0_(*δ*) + *α* (*δ* − *δ*_*c*_)*m*^2^ + 1/2 *βm*^4^, *α* > 0, *β* > 0, where *f*(*δ*) is the free energy functional, *δ* is the infinitesimal displacement of atoms (Fig. 3h and i), *m* is the order parameter, *α* and *β* are parameters. When *δ* > *δ*_*c*_, the phase has one energy state revealing one minimum (RBM or *G*-mode, *i.e*., all atoms in the wall of SWNTs have a synchronous radial or tangential vibration, respectively) (Fig. 3a), while the phase has two energy states revealing two minima (RTM) (Fig. 3a’) when *δ* < *δ*_c_.

**Figure 4:**
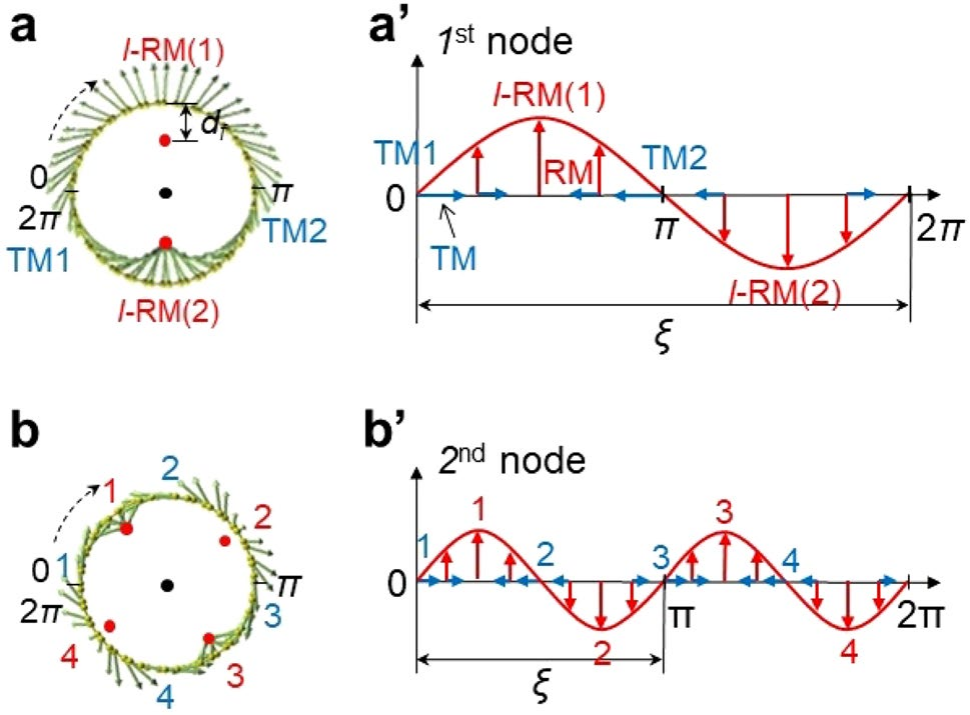
Conceptual explanation of RTMs and variation of depth of focus with node. (**a**, **b**) 1st and 2nd node RTMs for the concentric tube structure. The red solid dots indicate the decentered focus of Eigenvectors of *l*-RM. *d*_f_ is a depth of focus, the distance between the focus and the tube wall. The black dot in (**a**, **b**) indicates a focus of RBM where the depth of focus is *r*. (**a’**, **b’**) Sine curves of the RTMs where *ξ* is defined as a cycle of TM-RM-TM-RM-TM. Red and blue arrows indicate radial and transverse components deconvoluted from the slow varying Eigenvectors, respectively. Red and blue numbers in a-b’ indicate the number of *l*-RM and *l*-TM, respectively.

**Figure 5:**
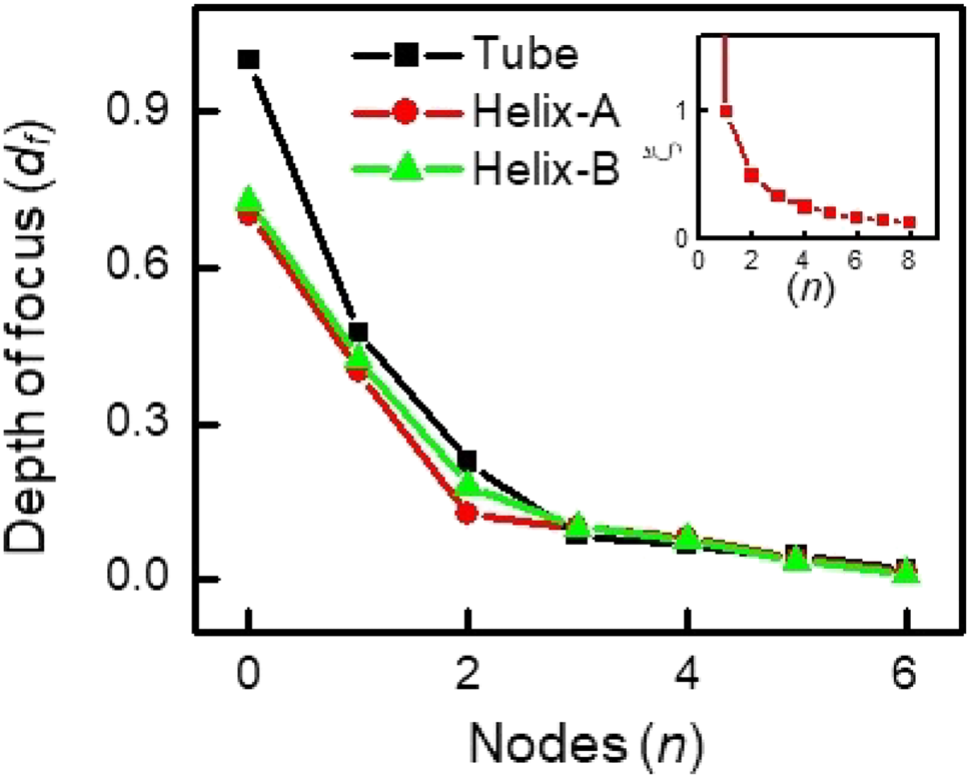
Variation of the depth of the focus of *l*-RMs with a node. Inset shows a variation of the coherence length with a node.

Figure 4 elucidates 1st and 2nd node RTMs featured by *l*-RMs and *l*-TMs. Both RTMs reveal two and four decentered focuses, respectively (red dots in Fig. 4a and b). Figure 4a’ and b’ depict the spatial variation of Eigenvectors, which is represented by a sine curve, *A*_*0*_*sin(ωt* + *φ),* where *A*_*0*_ is the amplitude, *ω* = 2π*f* (*f* is the angular frequency of oscillation), *t* is the time period, and *φ* is the phase (Fig. 4a’ and b’). The sine curve is regulated by the coherence length (*ξ*), a cycle of TM-RM-TM-RM-TM variation (the distance moved by the wave per one oscillation), which is described as *2πr/n* where* r* and *n* are a radius of the tubular structures and a number of nodes, respectively. With an increase in nodes, the coherence length, which is an intrinsic characteristic of the geometry, shortens, and the amplitude (*A*) of the sine curve (corresponding to pure radial components of *l*-RMs) decreases. The depth of focus (*d*_*f*_) of the *l*-RMs (Fig. 4a) also decreases with the nodes, as shown in Fig. 5. This indicates that the coherence length, the amplitude as well as the depth of focus (*d*_*f*_) can be indicators to estimate the intensity of the RTMs.

With the simulation data and their analysis (Figs. 3, 4, 5), we assign the strong peak at 169 cm^−1^ and its shoulder peak at 188 cm^−1^ for arc-SWNTs (Fig. 2) to the RBM (*l*-RBM) and the 1st node RTM, respectively. For Tuball-SWNTs, we assign 149 cm^−1^ and ~ 166 cm^−1^ peaks to RBM (*l*-RBM) and 1st node RTM respectively. We also assign many signals below ~ 500 cm^−1^ in the zoom-in spectra (Fig. 2b) to RTMs, 299 and 415 cm^−1^ peaks of Tuball-SWNTs to the 2^nd^ and the 3^rd^ node RTMs respectively, 343 cm^−1^ peak of Arc-SWNTs to the 2^nd^ node RTM. On the other hand, we could not assign the higher-frequency RTMs at 600–1500 cm^−1^. With the variation tendency of the RTMs with nodes (Fig. 3), we expect that the higher-frequency TM may dominate RTMs, and their intensity may gradually increase up to *G*-mode (which is contrary to the case of the lower-frequency RTMs below ~ 600 cm^−1^), except for the *D* bands at 1319 and 1340 cm^−1^.

The RBM peak at 149 cm^−1^ for Tuball-SWNTs corresponds to the diameters of ~ 2.2 nm on simulation (Fig. 3), which is at least ~ 10% overestimated, compared with the diameters measured to be 1.4–2.0 nm by HRTEM (Fig. 1). In this extension, we infer the weak peaks at 120 cm^−1^ and 137 cm^−1^ (in lower frequency than that of the RBM) corresponding to ~ 2.5 nm diameter to be from non-tubular carbon structures, *i.e.*, curved graphene^14^ with a curvature milder than the SWNTs of ~ 2.0 nm in diameter. Indeed, graphene structures coexist in the sample (Fig. 1b), and they can reveal *l*-RBM^15^ as well as RTMs when nano-curved (Fig. 3f).

We attribute two different Landau levels of RTMs for the opened tube structures (Fig. 3d and e) to the pseudo magnetic field. It has been reported that curved graphene produces a pseudo magnetic field due to strain^40,41^. This may explain the inconsistent RTMs on the Raman spectra (Fig. 2), further supporting the helix model of SWNTs. A helical SWNT possesses dangling carbon atoms at the asymmetric helical edges^37^, which break the rotational symmetry adding uneven strain to the system, resulting in diverse and inconsistent RTMs (Fig. 2b). Indeed, helical evidence is evident in Tuball-SWNTs (Fig. 1b) as well as arc-SWNTs^28^. The helical edges are vulnerable to environmental conditions, bundled or individual and freestanding or deposited on a substrate, explaining further the diversity of the low-frequency Raman signals for SWNTs reported^1–5,17–27^. The analysis may also explain the excitation wavelength dependence of the low-frequency Raman signals^2,5,23–25^ due to diverse curvatures of the helical edge (Supplementary Fig. 3), which can react differently with the energy range of the incident laser. Indeed, helical evidence of SWNTs, such as locally unrolled, twisted, or folded HRTEM morphologies, has been reported^30–33^. We expect that the ordered optical transitions (~ 150–250 cm^−1^) observed from 200 different isolated or bundled SWNTs reported by Paulo et al.^6^, the steplike dispersions (600–1100 cm^−1^) observed from two different bundled samples (arc-SWNTs and HiPco) reported by Fantini et al.^23^, as well as ZA-derived phonons (~ 400, ~ 800, and ~ 950 cm^−1^) observed from highly aligned SWNTs reported by Vierck et al.^27^ are the revelation of our Landau regulations of the RBMs and IFMs. Luo et al*.* also reported the steplike appearance of Raman signals in the energy range of 100–600 cm^−1^ observed from HiPco samples^24^.

We suggest the role of the RTMs interlinking the strongest *G*-mode and the 2^nd^ strong RBM of SWNTs in a slowly varying manner. This explains the symmetric Raman spectra of SWNTs in intensity between the RBM and the *G*-mode^1–5,19–27^. The spatial variation of the Eigenvectors on RTMs of SWNTs resembles the variation of vector fields from the divergence (comparable to RBM) to anti-symmetric curl (comparable to *G*-mode), as depicted in Fig. 3g. This suggests that our approach to Raman modes based on Landau theory can be extended to explain the transition of the vector field.

In summary, we manifested RTMs to be active Raman modes of SWNTs. They appear as ripple-like peaks between the RBM (~ 150 cm^−1^) and the *G*-mode (1592 cm^−1^), interlinking gradually. Our finding completes the analysis of the unique Raman spectrum for SWNTs where the ripple signals of SWNTs appearing at ~ 200–1440 cm^−1^ have been misinterpreted or disregarded for the last 30 years. The diversity of RBM and RTM peaks in shape and intensity (or absence) could be explained with the helix model of SWNTs, where the RBM becomes *l*-RBM. We propose that Landau theory can be a further way of understanding the nature of nanoscale matters with simple as well as powerful Raman analysis.

## Method

### TEM and Raman analysis

We analyzed two kinds of purified commercial SWNTs, Tuball (Oscial, Russia) and ASA-100F (arc-discharge, Hanwha Chemical, South Korea), where the purity is 80 wt% and 95 wt%, respectively. The impurity in Tubull samples includes metal nanoparticles as well as other carbonous structures (Fig. 1)^38^. Raman measurements were carried out using Renishaw In-Via Raman Microscope with laser excitation of 532 nm, a spot size of 1–2 μm, laser power of 5 mW, and powder density of 1.6 mW/µm^2^. Tuball samples were analyzed by Cs-corrected transmission electron microscope (Libra 200 HT Mc, Carl Zeiss). Earlier, we explored the arc-discharge sample as a helical structure with intensive high-resolution TEM observation^28^.

### Raman simulation

**Figure 6:**
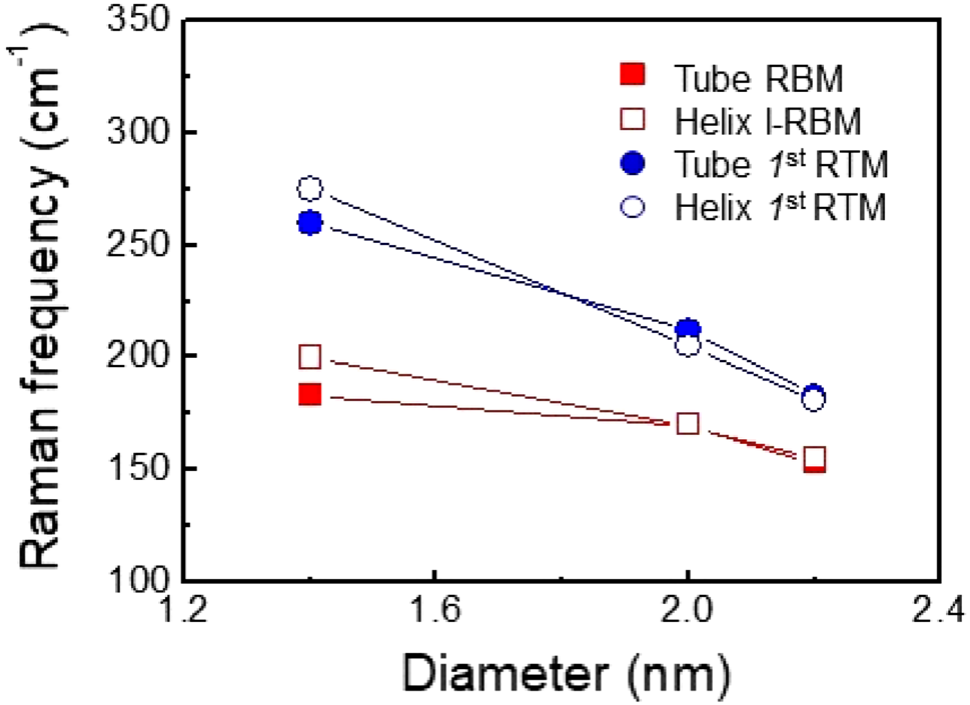
Simulated Raman frequency with the diameter of concentric and opened SWNTs. The data were obtained from the models with diameters of ~ 1.4, ~ 2.0, and ~ 2.2 nm (Supplementary Fig. 3).

We carried out the simulation with concentric tubes ((10, 10), (15, 15), and (16, 16) tubes), opened tubes (without distortion), distorted opened tubes, as well as curved graphene. For the distorted opened tube structures, we mimicked the shape of the helical SWNTs^37^. We used density functional theory (DFT) as implemented in the QUANTUM ESPRESSO simulation package^43^. Generalized gradient approximation (GGA) was used for the exchange–correlation energy of electrons and ultra-soft pseudopotentials to represent the interaction between ionic cores and valence electrons^44^. Kohn Sham wave functions were represented with a plane wave basis with an energy cutoff of 40 Ry, and a charge density cutoff of 240 Ry^45^ Integration over the Brillouin zone (BZ) was sampled with a mesh of 1 × 1x2 grid^46^. Dynamical matrices at the Γ point (q = 0) in BZ were computed using the perturbative linear response approach used in DFT (Fig. 6).

## Supplementary Information

Supplementary Information.

## Data Availability

The data that support the findings of the study are available in the public domain. However, the authors will be happy to discuss an appropriate accommodation for the reasonable request to J. L.
