# Supplementary material for: Radial-tangential mode of single-wall carbon nanotubes manifested by Landau regulation: reinterpretation of low- and intermediate-frequency Raman signals
Source: Sci Rep. 2023 Mar 27;13:5012. doi: 10.1038/s41598-023-32018-4 (PMC10042836; doi:10.1038/s41598-023-32018-4)
Supplement: Supplementary file 1 — Supplementary Information. [file 41598_2023_32018_MOESM1_ESM.pdf]

Supporting Information

Radial-Tangential Mode Manifested by Landau Regulation: Reinterpretation of Low Energy Raman Signals of Single-Wall Carbon Nanotubes

K. P. S. S. Hembram[1], Jin-Gyu Kim[2], Sang-Gil Lee[2], Jeongwon Park[3,4], and Jae-Kap Lee[*1]

[1]Center for Opto-Electronic Materials and Devices, Korea Institute of Science and Technology (KIST), Seoul 02792, Republic of Korea

[2]Division of Electron Microscopic Research, Korea Basic Science Institute, Daejeon 305-333, Korea

[3]Department of Electrical and Biomedical Engineering, University of Nevada, Reno, NV 89557, USA

[4]School of Electrical Engineering and Computer Science, University of Ottawa, Ottawa, ON K1N6N5, Canada.

This PDF file includes the following:

-Figs. S1 to S3

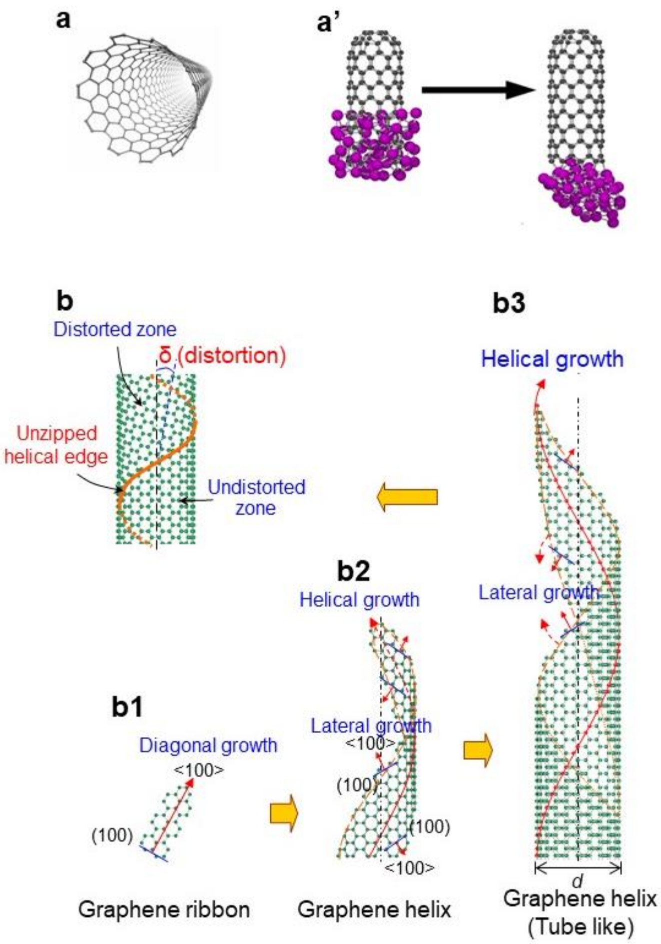

Fig. S1. Two structure models for SWNTs. a, Regular tube model for SWNTs, resulted from tubular growth of SWNT nuclei (a'). This conventional model was derived from the structure model of multi-wall carbon nanotubes. **b**, Helix model for SWNTs, resulted from helical growth of graphene nanoribbons (b1-b3). The model was suggested by Lee *et al*.[28] in 2014. In the helix model, a graphene nanoribbon is a nucleus (b1) that spirally and laterally grows, resulting in tube-like graphene structures. The materials can be seen as perfect tubes, defective tubes, and nodal tubes, according to the degree of the helical scroll, and generally appear to be locally distorted (see Figure S2). In the helix model, the chiral theory of SWNTs is not a necessary condition, and the chirality is interpreted as distortion (δ) (b).

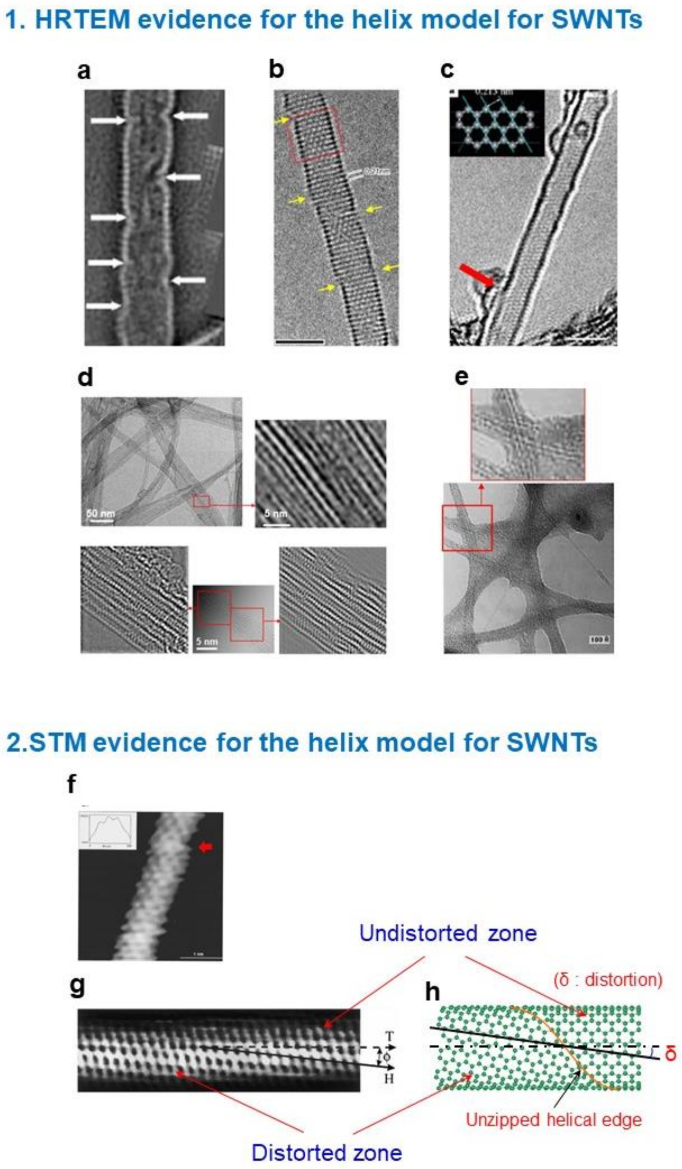

Fig. S2. HRTEM and STM evidence for the helix model of SWNTs. **a**, Meyer *et al*.'s HRTEM image,[29] revealing evidence of helical SWNT. They reported the diameter of the samples used to be 1.46~1.63 nm. **b**, Suenaga *et al*.'s HRTEM image.[30] Yellow arrows indicate the traces of helical SWNT. Scale bar=3 nm. **c**, Hashimoto *et al*.'s HRTEM image.[31] The red arrow indicates a disconnected lattice. The tubule is bumpy. These support the helical hypothesis of SWNTs. Scale bar, 2 nm. **d**, Lee *et al*.'s HRTEM images.[28] **e**, Bethune *et al*.'s HRTEM image,[32] revealing nodal morphology, evidence of the helical structure model of SWNTs. **f**, Ge *et al*.'s STM image.[34] Red arrows indicate the distorted zone of the tubule, which is evidence of the helical structure model of SWNTs. **g**, Wildöer, *et al*.'s STM image.[35] **h**, New analysis for the STM image (g) in terms of the helix model. The chirality (*φ*) based on the concentric

tube model (g) is interpreted as distortion δ (due to helical growth) in the helix model (h).

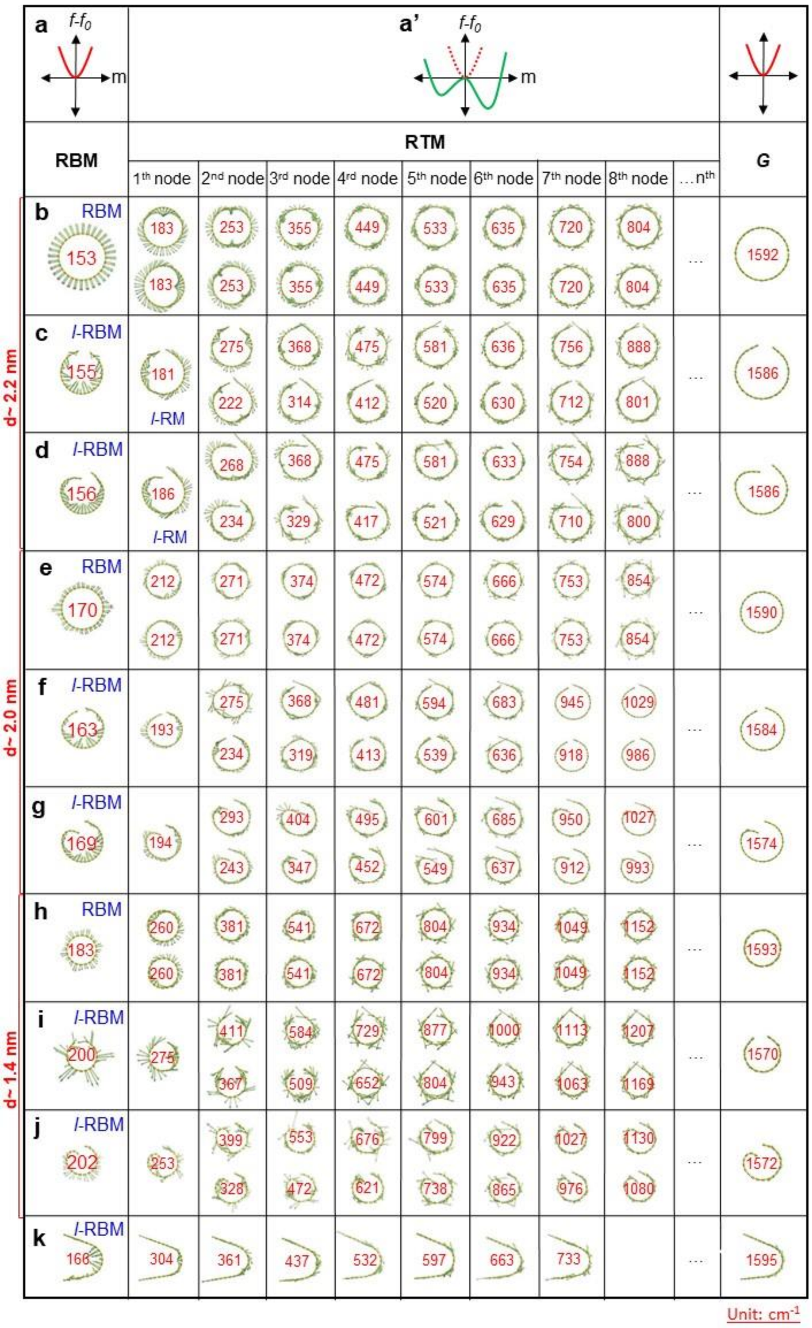

Fig. S3. Simulated active-Raman spectra of different SWNT structures. a,**a'**, Landau free energy landscape. **b-d**, Simulated active-Raman spectra for regular tube (b) and opened (c,d) SWNTs with ~2.2 nm in diameter. **e-g**, Simulated active-Raman spectra for regular tube (e) and opened (f,g) SWNTs with ~2.0 nm in diameter. **h-j**, Simulated active-Raman spectra for regular tube (h) and opened (i,j) SWNTs with ~1.4 nm in diameter. **k**, Simulated active-Raman spectra for curved graphene structure. The red numbers in (b-k) represent the frequency of the modes.
